# Phytochemical Characterization, Bioactivities, and Nanoparticle-Based Topical Gel Formulation Development from Four *Mitragyna speciosa* Varieties

**DOI:** 10.3390/gels11070494

**Published:** 2025-06-26

**Authors:** Pimporn Anantaworasakul, Weeraya Preedalikit, Phunsuk Anantaworasakul, Sudarshan Singh, Aekkhaluck Intharuksa, Warunya Arunotayanun, Mingkwan Na Takuathung, Songwut Yotsawimonwat, Chuda Chittasupho

**Affiliations:** 1 Faculty of Pharmacy, Chiang Mai University, Chiang Mai 50200, Thailand; pimporn.a@cmu.ac.th (P.A.); sudarshan.s@cmu.ac.th (S.S.); aekkhaluck.int@cmu.ac.th (A.I.); songwut.y@cmu.ac.th (S.Y.); 2 Department of Cosmetic Sciences, School of Pharmaceutical Sciences, University of Phayao, Phayao 56000, Thailand; weeraya.pr@up.ac.th; 3 Regional Medical Sciences Center 2 Phitsanulok, Phitsanulok 65000, Thailand; phunsuk.a@dmsc.mail.go.th; 4 Office of Research Administration, Chiang Mai University, Chiang Mai 50200, Thailand; 5 Kanchanabhishek Institute of Medical and Public Health Technology, Faculty of Public Health and Allied Health Science, Praboromarajchanok Institute, Nonthaburi 11150, Thailand; warunya@kmpht.ac.th; 6 Department of Pharmacology, Faculty of Medicine, Chiang Mai University, Chiang Mai 50200, Thailand; mingkwan.n@cmu.ac.th; 7 Clinical Research Center for Food and Herbal Product Trials and Development (CR-FAH), Faculty of Medicine, Chiang Mai University, Chiang Mai 50200, Thailand

**Keywords:** topical gel, poloxamer micelles, antioxidant activity, anti-aging activity, cosmeceutical formulation

## Abstract

*Mitragyna speciosa* (kratom) is a traditional medicinal plant rich in bioactive alkaloids and phenolics, known for their antioxidant and anti-aging properties. This study aimed to develop nanoparticle-based topical gels from ethanolic extracts of four kratom varieties, including Kan Daeng (KD), Hang Kang (HK), Tai Bai-yao (KY), and Kan Keaw (KG). Kratom NPs were prepared using a solvent displacement method. The resulting nanoparticles (NPs) exhibited sizes of 201.9–256.2 nm, polydispersity indices (PDI) below 0.3, and a zeta potential between −22.6 and −29.6 mV. The phytochemical analysis revealed that KG and KY extracts contained the highest total phenolic content (TPC) and total flavonoid content (TFC), which were mostly retained after NP formulation. The HPLC analysis confirmed HK as the richest source of mitragynine (9.97 ± 0.10% *w*/*w*), while NP formulations displayed slightly reduced levels. Antioxidant activities assessed by DPPH, ABTS, and FRAP assays revealed enhanced radical scavenging in nanoparticle formulations, with IC_50_ values ranging from 151.23 to 199.87 µg/mL (DPPH) and 207.37 to 272.83 µg/mL (ABTS). All formulations exhibited a significant inhibition of collagenase (80.56 ± 1.60 to 97.23 ± 0.29%), elastase (45.46 ± 6.53 to 52.19 ± 1.20%), and hyaluronidase (83.23 ± 2.34 to 91.67 ± 3.56%), with nanoparticle forms showing superior enzyme inhibition. Notably, nanoparticle formulations exhibited superior inhibitory effects compared to crude extracts. HaCaT cytotoxicity tests confirmed high biocompatibility (IC_50_ > 700 µg/mL), especially for KD and KG NPs. The NP-loaded gels demonstrated acceptable physicochemical stability after heating/cooling cycle testing, with pH (7.27 to 7.88), viscosity (10.719 to 12.602 Pa·s), and favorable visual and textural properties. In summary, KG and KY cultivars emerged as the most promising cosmeceutical candidates due to their superior phytochemical content, antioxidant capacity, enzyme-inhibitory activities, and formulation performance. These findings support the potential use of KG NP and KY NP-loaded gels as multifunctional cosmeceutical agents for antioxidant protection, anti-aging, and skin rejuvenation.

## 1. Introduction

Skin aging is a multifactorial biological process influenced by intrinsic factors such as genetics and hormonal changes, as well as extrinsic factors like ultraviolet radiation, pollution, and oxidative stress [1]. One of the primary contributors to this process is the overproduction of reactive oxygen species (ROS), which leads to oxidative damage and the activation of matrix-degrading enzymes such as collagenase, elastase, and hyaluronidase [2,3]. One of the primary contributors to this process is the overproduction of reactive oxygen species (ROS), which can originate from sources such as UV radiation, environmental pollution, and mitochondrial dysfunction, resulting in oxidative damage and the activation of matrix-degrading enzymes. These enzymes include collagenase, which targets collagen fibers; elastase, which degrades elastin; and hyaluronidase, which breaks down hyaluronic acid, all of which contribute to the deterioration of skin structure and function [4]. These enzymes degrade extracellular matrix (ECM) components, resulting in skin wrinkling, loss of elasticity, and dryness. Consequently, the development of topical agents with antioxidant properties and the ability to inhibit collagenase, elastase, and hyaluronidase activity has gained significant attention in dermatological and cosmeceutical research.

Kratom (*Mitragyna speciosa* (Korth.) Havil.), a tropical medicinal plant native to Southeast Asia, has long been used in traditional medicine for its analgesic, anti-inflammatory, hypolipidemic, and energetic effects [5,6,7,8]. Recent studies have reported that kratom leaves contain a rich variety of phytochemicals, including polyphenols, flavonoids, and alkaloids such as mitragynine, which exhibit potent antioxidant and anti-inflammatory activities [9]. Given its antioxidant and enzyme-inhibitory effects, these properties suggest that kratom holds promise as a natural ingredient in anti-aging skincare products by helping to protect against oxidative stress and extracellular matrix degradation in the skin. However, its practical application in topical formulations is limited due to poor water solubility, physicochemical instability, and limited skin dermal permeability [10].

Nanoparticle-based delivery systems have emerged as a promising strategy to overcome the limitations of conventional plant extract formulations. Formulating plant-derived bioactive compounds into nanoparticulate form can enhance their solubility, stability, and bioavailability [11,12]. Among various nanocarriers, poloxamer-based micelles are particularly attractive due to their excellent biocompatibility and capacity to improve the dermal delivery of hydrophobic phytoconstituents. Poloxamers, also known as Pluronics, consist of hydrophilic polyethylene oxide (PEO) and hydrophobic polypropylene oxide (PPO) blocks arranged in a triblock configuration (PEO–PPO–PEO). In aqueous environments, these molecules self-assemble into micelles with a hydrophobic PPO core and a hydrophilic PEO corona, enabling the encapsulation of poorly water-soluble compounds [13]. In addition to the solubilization capacity, poloxamer micelles exhibit thermoresponsive behavior, forming gels upon temperature elevation due to micelle packing and network formation, which is an advantageous feature for controlled drug release and enhanced skin adhesion [14]. Furthermore, the presence of the PEO corona provides steric stabilization, reducing particle aggregation and contributing to the long-term colloidal stability of the formulation [15]. These properties make poloxamer-based micelles particularly suitable for the delivery of hydrophobic phytoconstituents in topical applications. In this study, poloxamer-based micellar nanoparticles were employed to encapsulate various kratom extracts (KD, HK, KY, and KG), with the aim of improving their aqueous solubility, enhancing biological activities, and thereby increasing their cosmeceutical potential. We hypothesize that kratom extracts from different cultivars, when encapsulated into poloxamer-based micellar NPs, will retain or enhance their antioxidant and enzyme-inhibitory activities, while improving biocompatibility and formulation stability. This nanoparticle-based delivery system is expected to provide a multifunctional platform suitable for cosmeceutical skincare applications.

## 2. Results and Discussion

### 2.1. Characterization of Kratom Nanoparticles

#### 2.1.1. Particle Size and Polydispersity Index (PDI)

The dynamic light scattering (DLS) analysis confirmed that all kratom nanoparticle (NP) formulations achieved nanoscale dimensions, with mean particle sizes ranging from 201.90 ± 0.69 nm to 256.20 ± 2.68 nm. Among the four formulations, KG NP exhibited the smallest particle size (201.90 ± 0.69 nm), followed by KY NP (221.00 ± 0.60 nm), HK NP (247.70 ± 12.20 nm), and KD NP (256.20 ± 2.68 nm). The blank nanoparticle system displayed a notably smaller particle size of 113.50 ± 0.87 nm, likely due to the absence of encapsulated phytochemicals.

All formulations demonstrated PDI values below 0.3, reflecting narrow and uniform size distributions, which are considered optimal for pharmaceutical nanoparticle systems. The lowest PDI was observed in KY NP (0.097 ± 0.013), followed by KG NP (0.183 ± 0.007), HK NP (0.188 ± 0.030), and KD NP (0.262 ± 0.006). The blank formulation had a PDI of 0.211 ± 0.010. These results meet the accepted criteria for monodisperse nanoparticles, where particle size uniformity is critical for reproducible skin permeation and consistent therapeutic performance. Differences in size and PDI may reflect intrinsic phytochemical variations among kratom varieties.

#### 2.1.2. Zeta Potential Values

Zeta potential measurements revealed that all kratom NPs formulations possessed negative surface charges, ranging from −22.60 ± 1.47 mV to −29.57 ± 3.23 mV. KY NP demonstrated the most negative zeta potential (−29.57 ± 3.23 mV), followed closely by HK NP (−29.40 ± 3.05 mV), KD NP (−26.20 ± 0.38 mV), and KG NP (−22.60 ± 1.47 mV). The blank formulation exhibited a ZP of −23.30 ± 3.32 mV. Typically, NPs with zeta potential values greater than ±30 mV are often considered highly stable due to strong electrostatic repulsion. NPs within the −20 to −30 mV range may still maintain colloidal stability, particularly when formulated with steric stabilizers. In this study, the use of a poloxamer likely provided additional steric hindrance via its hydrophilic polyethylene oxide chains, forming a protective hydration shell that prevented particle aggregation. This dual stabilization mechanism—electrostatic and steric—is consistent with the previous findings where poloxamer coatings contributed to the stability of nanoparticles, even with moderately negative zeta potentials [16]. Together, the observed zeta potential values and the presence of steric stabilizers suggest that all prepared nanoparticles possess adequate colloidal stability for topical delivery.

##### Total Phenolic Content

**Figure 1:**
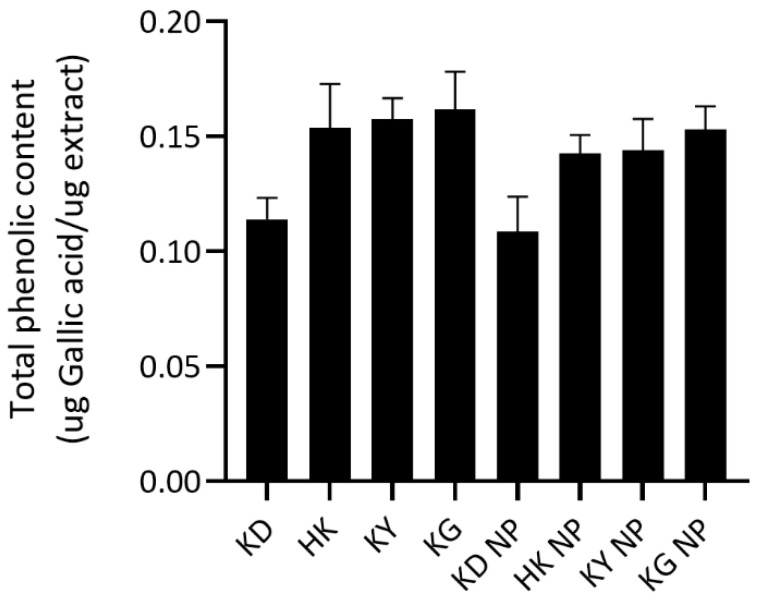
Total phenolic content (TPC) of kratom leaf extracts and their corresponding NP formulations. Data are presented as mean ± SD (*n* = 3). No statistically significant differences were observed between each extract and its corresponding nanoparticle formulation (*p* > 0.05).

The total phenolic content (TPC) of each extract and its corresponding NPs formulation was quantified and expressed in micrograms of gallic acid equivalents per microgram of extract (µg GAE/µg extract) (Figure 1). Among the crude extracts, KG exhibited the highest TPC (0.1616 ± 0.0164 µg GAE/µg extract), followed by KY (0.1576 ± 0.0089), HK (0.1537 ± 0.0192), and KD (0.1138 ± 0.0094). A similar trend was observed in NP formulations, with the highest TPC observed in KG NP (0.1529 ± 0.0102), followed by KY NP (0.1439 ± 0.0137), HK NP (0.1426 ± 0.0079), and KD NP (0.1084 ± 0.0154). The statistical analysis revealed that KD had a significantly lower TPC compared to HK, KY, and KG, as well as significantly lower than KG NP.

These findings highlight the superior phenolic content of KG and KY varieties, supporting their traditional use and potential antioxidant efficacy. In contrast, the lower TPC in KD suggests a reduced contribution to phenolic-dependent antioxidant activity. Notably, no statistically significant differences in TPC were observed between each crude extract and its corresponding NP formulations (*p* > 0.05). This suggests that a poloxamer-based encapsulation system effectively preserves the phenolic constituents without causing degradation or loss. It also indicates that the phenolic compounds were either not significantly retained within the micelle core or remained fully accessible or reactive with the Folin–Ciocalteu reagent following encapsulation.

##### Total Flavonoid Content

**Figure 2:**
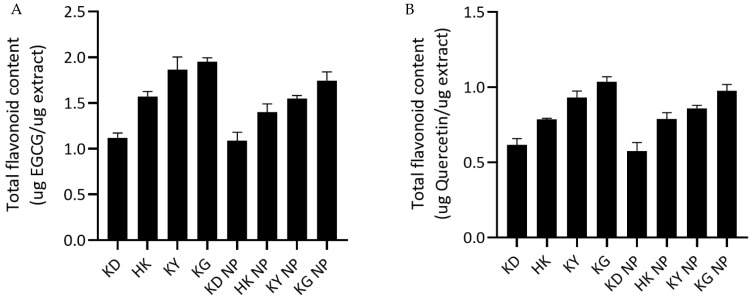
Total flavonoid content (TFC) of kratom leaf extracts and their corresponding NP formulations. (**A**) TFC expressed as epigallocatechin gallate equivalents (µg EGCG/µg extract). (**B**) TFC expressed as quercetin equivalents (µg quercetin/µg extract). Data are shown as mean ± SD (*n* = 3). No statistically significant differences were observed between each extract and its corresponding NP formulation (*p* > 0.05).

The total flavonoid content (TFC), quantified using both EGCG and quercetin equivalents, was assessed for each kratom extract and its corresponding poloxamer-based NP formulation (Figure 2). Among the crude extracts, KG exhibited the highest TFC, with values of 1.9539 ± 0.0413 µg EGCG equivalent/µg extract and 1.0370 ± 0.0322 µg quercetin equivalent/µg extract, followed by KY and HK. In contrast, KD showed the lowest TFC, with values of 1.1199 ± 0.0524 and 0.6153 ± 0.0431 EGCG and quercetin equivalents per µg extract, respectively. The NP formulations mirrored these trends: KG NP and KY NP retained the highest TFC values, while KD NP consistently showed the lowest. The statistical analysis indicated no significant differences in TFC between each extract and its corresponding nanoparticle formulation (*p* > 0.05), suggesting that the NP preparation process effectively preserved the flavonoid content of the extracts.

##### Quantification of Mitragynine in Extracts and Nanoparticle Formulations

**Figure 3:**
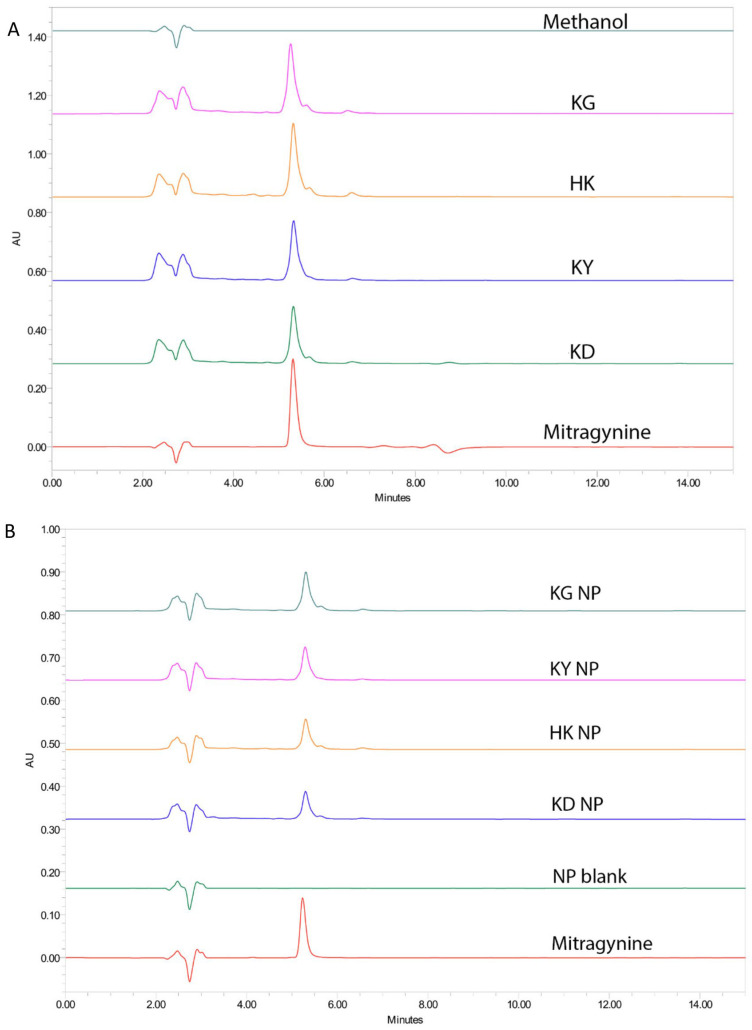
Representative HPLC chromatograms of mitragynine in kratom samples (**A**), crude extracts (KD, HK, KY, KG), and mitragynine standard (40 ppm), and (**B**) corresponding NP formulations and blank formulation (NP blank). Mitragynine was consistently detected at a retention time of 5.2–5.3 min.

High-performance liquid chromatography (HPLC) was used to quantify mitragynine in various kratom extracts and their corresponding NP formulations. Under the selected chromatographic conditions, the retention time (RT) of mitragynine was consistently observed at approximately 5.2–5.3 min. Representative chromatograms included samples from crude extracts (KD, HK, KY, and KG), NP formulations (KD NP, HK NP, KY NP, and KG NP), a blank nanoparticle formulation, and a mitragynine standard (40 ppm) (Figure 3). Each sample showed a sharp peak corresponding to mitragynine. The absence of a corresponding peak in the blank nanoparticle formulation confirmed the specificity of the method and the absence of mitragynine contamination from excipients.

The tailing observed in the chromatograms of nanoparticle (NP) formulations may be attributed to the interaction of the mitragynine with the poloxamer, which can cause delayed elution and result in peak asymmetry [17].

The mitragynine content in the crude extracts and corresponding NP formulations was quantified and expressed as both % *w*/*w* and mg/g of sample. Among the crude extracts, HK exhibited the highest mitragynine content (9.97 ± 0.10% *w*/*w*; 98.39 ± 1.05 mg/g), followed by KG (9.64 ± 0.24%, 96.41 ± 0.24 mg/g), KY (7.65 ± 0.13%, 76.52 ± 0.10 mg/g), and KD (7.48 ± 0.18%, 74.82 ± 0.14 mg/g). Among the NPs, KG NP retained the highest mitragynine content (8.20 ± 0.17% *w*/*w*; 81.97 ± 0.17 mg/g), followed by KY NP (6.27 ± 0.22%; 62.75 ± 0.22 mg/g), HK NP (5.82 ± 0.15%; 58.25 ± 0.15 mg/g), and KD NP (5.15 ± 0.24%; 51.51 ± 0.24 mg/g).

The calibration curve for the quantification method demonstrated excellent linearity over the concentration range of 10–100 µg/mL, with a correlation coefficient (R^2^) of 0.9987. The linear regression equation was y = 30,629x + 166,812. The method exhibited good accuracy, with low residuals across all concentrations. The residual standard deviation was calculated to be 39,008.53, which was subsequently used to determine the sensitivity of the method. The limit of detection (LOD) and limit of quantification (LOQ) were calculated based on the ICH Q2 (R1) guideline as 4.20 µg/mL and 12.74 µg/mL, respectively, indicating the method’s suitability for detecting and quantifying the analyte within the working range.

The precision analysis displayed low relative standard deviation (RSD) values for all NP samples, indicating excellent analytical repeatability: KG NP (0.20%), HK NP (0.26%), KY NP (0.34%), and KD NP (0.46%). All values remained within acceptable limits for bioanalytical methods. These findings confirm that HK and KG extracts contain the highest levels of mitragynine, supporting their potential as potent sources of bioactive alkaloids. Although the mitragynine content decreased following nanoparticle formulation, KG NP and KY NP still retained relatively high concentrations, suggesting good formulation efficiency and compound stability during the encapsulation process.

The encapsulation efficiency (%EE) of mitragynine in the nanoparticle formulations varied depending on the kratom source. The KD NP formulation showed an average %EE of 79.25 ± 0.49%, while KY NP exhibited a significantly higher encapsulation efficiency of 94.34 ± 0.36%. In contrast, HK NP presented the lowest %EE among the four, with an average of 67.76 ± 0.66%. Notably, the KG NP formulation demonstrated the highest %EE at 97.78 ± 0.13%, indicating a highly efficient encapsulation. These results suggest that the phytochemical profile of different kratom sources may influence the encapsulation capacity of the nanoparticles. 

##### DPPH Radical Scavenging Activity

**Figure 4:**
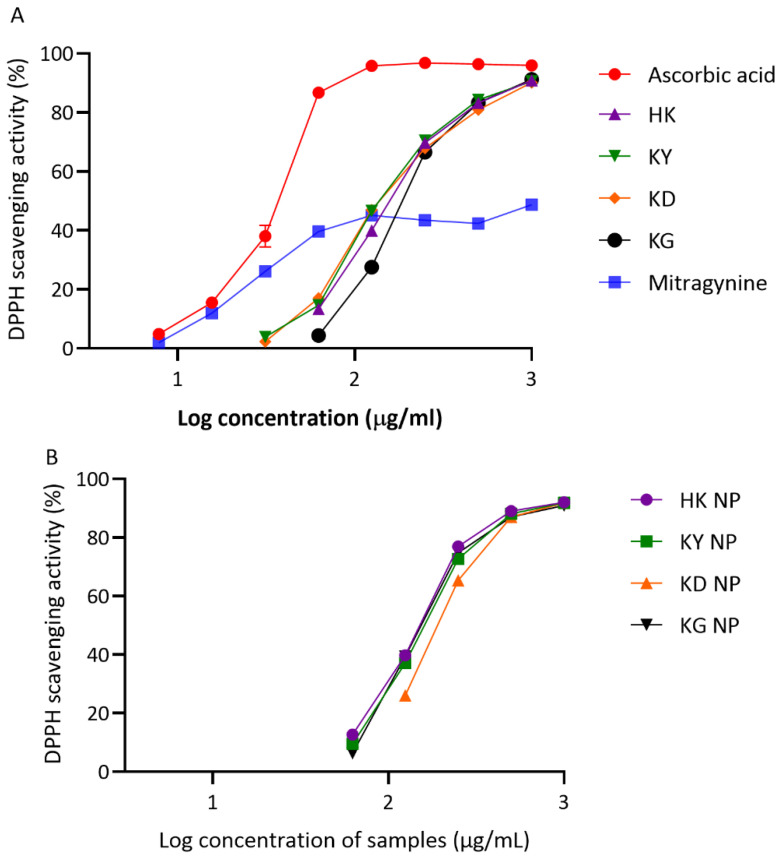
DPPH radical scavenging activity of kratom extracts and their NP formulations. (**A**) Dose-dependent scavenging activity of crude extracts (HK, KY, KD, and KG) compared to ascorbic acid (a positive control). (**B**) Scavenging activity of NP formulations (HK NP, KY NP, KD NP, and KG NP). Data are shown as mean ± SD (*n* = 3).

The antioxidant activities of kratom leaf extracts and their corresponding NP formulations were evaluated using the DPPH radical scavenging assay. As illustrated in Figure 4A,B, all samples exhibited dose-dependent scavenging activity, though with varying degrees of potency. Among the crude extracts, KY showed the highest DPPH radical scavenging activity, with an IC_50_ of 152.47 ± 3.26 µg/mL, followed closely by KD (157.90 ± 1.65 µg/mL) and HK (167.60 ± 1.91 µg/mL). KG demonstrated the lowest antioxidant activity among the crude samples, with an IC_50_ of 197.70 ± 4.30 µg/mL. For the NP formulations, HK NP exhibited the strongest antioxidant capacity, with an IC_50_ of 151.23 ± 3.55 µg/mL, slightly outperforming its crude counterpart. KG NP and KY NP followed, with IC_50_ values of 160.17 ± 3.76 µg/mL and 163.97 ± 0.61 µg/mL, respectively. In contrast, KD NP showed a slightly reduced activity compared to its extract form, with an IC_50_ of 199.87 ± 2.18 µg/mL. This underperformance may be attributed to the specific phytochemical composition of the KD strain, which contained the lowest total phenolic and flavonoid content among the varieties studied. As expected, the positive control, ascorbic acid, demonstrated the strongest antioxidant activity, with an IC_50_ of 34.99 ± 1.47 µg/mL. In comparison, mitragynine—the major alkaloid in kratom—showed a relatively weak radical scavenging activity, with an IC_50_ of 602.60 ± 17.11 µg/mL, which was higher than that of all kratom samples tested.

##### ABTS Radical Scavenging Activity

**Figure 5:**
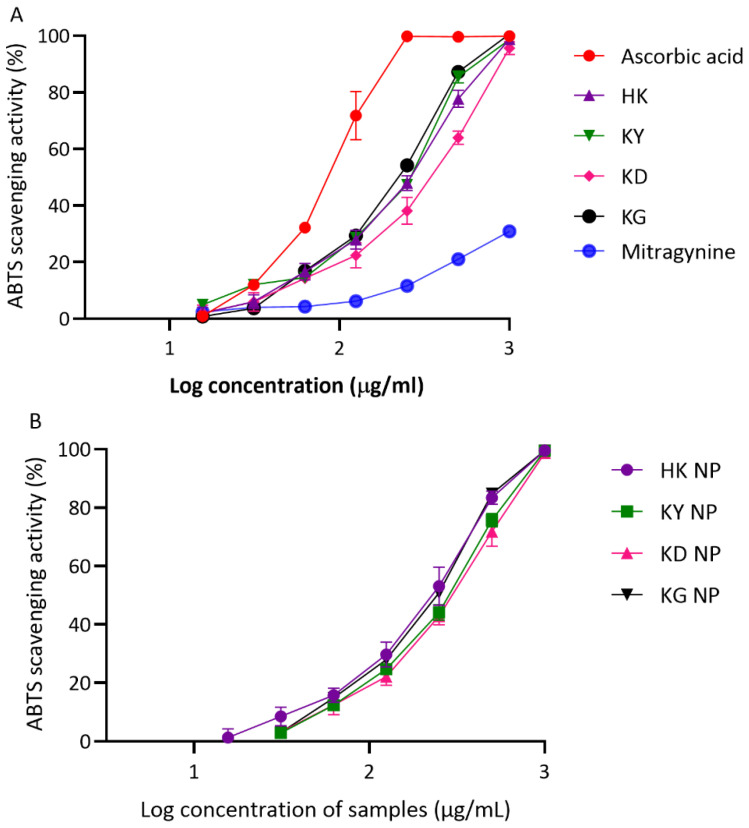
ABTS radical scavenging activity of kratom extracts and their NP formulations. (**A**) Antioxidant activity of crude extracts (HK, KY, KD, and KG) compared with ascorbic acid (positive control). (**B**) Antioxidant activity of NP formulations (HK NP, KY NP, KD NP, and KG NP). Results are presented as percentage inhibition of ABTS versus log concentration (µg/mL). Data are expressed as mean ± SD (*n* = 3).

The ABTS assay further confirmed the antioxidant potential of both kratom leaf extracts and their corresponding NP formulations (Figure 5A,B). In this assay, the IC_50_ values of the crude extracts followed a slightly different pattern compared to the DPPH results. KG showed the strongest activity among the extracts (201.67 ± 5.05 µg/mL), followed by KY (217.93 ± 5.53 µg/mL) and HK (229.77 ± 22.01 µg/mL). KD exhibited the weakest ABTS scavenging capacity, with an IC_50_ of 304.23 ± 38.52 µg/mL. The NP formulations generally retained or slightly enhanced antioxidant activity compared to their corresponding crude extracts. HK NP again showed the strongest activity among the NP samples (207.37 ± 28.26 µg/mL). KG NP and KY NP followed, with IC_50_ values of 216.40 ± 11.40 µg/mL and 255.63 ± 12.58 µg/mL, respectively. Interestingly, KD NP showed improved activity compared to its crude extract, with a lower IC_50_ of 272.83 ± 28.84 µg/mL As expected, ascorbic acid remained the most effective ABTS scavenger, with an IC_50_ of 83.16 ± 6.27 µg/mL. In contrast, mitragynine—the major alkaloid in kratom—showed minimal ABTS scavenging activity, with a markedly higher IC_50_ value of 2022.00 ± 108.53 µg/mL, confirming that it is not a major contributor to kratom’s antioxidant effect.

Overall, all kratom extracts possessed a moderate antioxidant capacity in both DPPH and ABTS assays. Among the extracts, KY and HK showed slightly stronger activities than KD and KG, consistent with their relatively higher total phenolic and flavonoid contents. The incorporation of the extracts into NP formulations generally preserved or modestly improved the antioxidant capacity, particularly in the ABTS assay. Notably, HK NP demonstrated the lowest IC_50_ values among NP formulations in both assays, suggesting the effective retention and stability of antioxidant constituents during formulation. Interestingly, mitragynine exhibited the weakest antioxidant activity, reinforcing that the observed effects are likely attributable to other phenolic or flavonoid constituents in kratom rather than this primary alkaloid. These findings support the hypothesis that kratom’s antioxidant potential is primarily driven by its phenolic and flavonoid compounds rather than by mitragynine. Ascorbic acid, used as a positive control, consistently demonstrated superior radical scavenging performance, underscoring the moderate nature of kratom’s antioxidant capacity.

##### Ferric Reducing Antioxidant Power (FRAP) Activity

**Figure 6:**
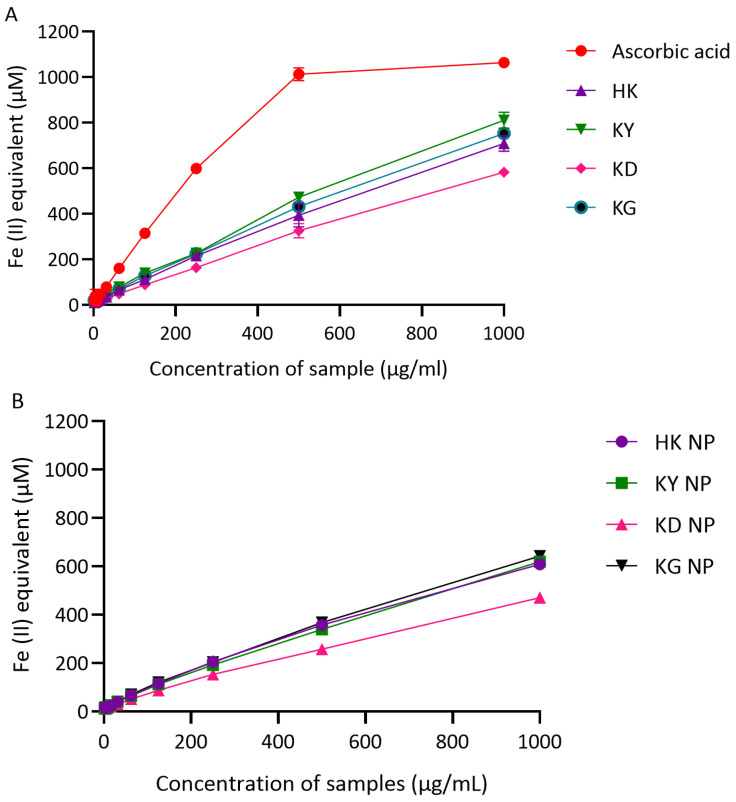
Ferric reducing power (FRAP) of kratom extracts and their corresponding NP formulations. (**A**) Reducing capacity of crude extracts (HK, KY, KD, and KG) compared with ascorbic acid (positive control), expressed as Fe(II) equivalents (µM). (**B**) Reducing capacity of NP formulations (KD NP, HK NP, KY NP, and KG NP). Results are shown as mean ± SD (*n* = 3).

The reducing power of kratom leaf extracts and their corresponding NP formulations was assessed using the ferric reducing antioxidant power (FRAP) assay, with ascorbic acid serving as a positive control. Results are expressed in terms of Fe(II) equivalents (µM), with higher values indicating a greater ferric reducing capacity. As shown in Figure 6A, ascorbic acid demonstrated the strongest reducing ability in a dose-dependent manner, reaching 1063.06 µM Fe(II) equivalents at 1000 µg/mL. Among the crude extracts, KY exhibited the highest FRAP value (810.55 µM), followed by KG (752.48 µM), HK (708.18 µM), and KD (582.61 µM), all measured at 1000 µg/mL. These results indicate that all kratom leaf extracts possess a notable ferric reducing activity, with KY and KG showing the strongest effects. In the NP formulations (Figure 6B), a similar trend of dose-dependent increase in FRAP activity was observed. At 1000 µg/mL, KG NP demonstrated the highest Fe(II) equivalent value (642.55 µM), followed by KY NP (619.12 µM), HK NP (608.65 µM), and KD NP (470.49 µM). These results suggest that the ferric reducing power was generally preserved after nanoparticle formulation, although slightly reduced compared to the crude extracts.

##### Correlation Between Phytochemical Content and Antioxidant Activities

A Pearson correlation analysis was conducted to evaluate the relationships between phytochemical content (TPC and TFC, measured as EGCG and quercetin equivalents) and IC_50_ values of crude kratom extracts (KD, HK, KY, KG) and their corresponding NP formulations (KD NP, HK NP, KY NP, KG NP) (Table 1).

Among the extract samples, a strong and statistically significant negative correlation was observed between TPC and ABTS IC_50_ (r = −0.9755, *p* = 0.0245), indicating that phenolic compounds play a key role in enhancing free radical scavenging. Similarly, TFC, whether measured as EGCG equivalents (r = −0.9856, *p* = 0.0144) or quercetin equivalents (r = −0.9856, *p* = 0.0144), also showed a strong and statistically significant negative correlation with ABTS IC_50_ values. These findings highlight flavonoids as potent contributors to hydrogen atom donation and radical neutralization. In contrast, correlations between the phytochemical content and DPPH IC_50_ values were weaker and not statistically significant. TPC showed a moderate positive correlation (r = 0.5802, *p* = 0.4198), while TFC (EGCG and quercetin) yielded similar trends (r ≈ 0.63, *p* > 0.37). The FRAP assay results demonstrated strong positive correlations between reducing power and both TPC (r = 0.8492, *p* = 0.1508) and TFC (r ≈ 0.87, *p* ≈ 0.13). These findings suggest that both phenolic and flavonoid compounds contribute meaningfully to the electron donation involved in ferric ion reduction.

In the NP formulations, similar trends were observed. TPC exhibited a strong negative correlation with ABTS IC_50_ (r = −0.9308, *p* = 0.0692), suggesting that the phenolic content retained in the NP formulations continues to contribute to the antioxidant capacity. Likewise, TFC (EGCG and quercetin) also showed strong negative correlations with ABTS IC_50_ (r = −0.86), supporting the role of flavonoids in radical scavenging even after nanoencapsulation. For the FRAP assay, all phytochemical parameters demonstrated strong positive correlations: TPC (r = 0.8566), TFC-EGCG (r = 0.9106), and TFC-quercetin (r = 0.9108), with *p*-values ranging from around 0.09 to 0.14. Although these correlations did not reach statistical significance, the trends clearly reflect that nanoparticle incorporation preserved the reducing potential associated with phenolic and flavonoid compounds. Correlations with DPPH were again weaker and not statistically significant (r = −0.79 for both TPC and TFC), reinforcing the notion that this assay may be less responsive to subtle phytochemical variations in both crude and nanoparticle-formulated systems.

The antioxidant performance of kratom extracts and their NP formulations can be largely attributed to their phenolic content. Strong positive correlations between total phenolic content (TPC) and both FRAP and ABTS assays suggest that phenolic compounds are the primary contributors to antioxidant activity, particularly in hydrophilic systems where electron donation and radical scavenging predominate. These assays, which operate in aqueous environments, effectively reflect the redox potential of polyphenols commonly present in kratom. In contrast, the DPPH assay, conducted in a less polar (absolute ethanol) medium, showed no significant correlation with TPC. This discrepancy may stem from the specific reactivity of certain phenolic compounds, which may be less efficient at quenching DPPH radicals or hindered by limited solubility or matrix effects in non-aqueous environments [18]. Therefore, while TPC is a strong predictor of antioxidant performance in ABTS and FRAP assays, it does not reliably predict DPPH scavenging under these conditions. Moreover, the consistently low correlation between total flavonoid content (TFC) and antioxidant activity across all assays indicates that flavonoids, although present, may not be the dominant bioactive constituents in these extracts. The reduced antioxidant capacity observed in most NP formulations may be due to partial encapsulation, which limits the accessibility of free phenolic compounds or from dilution effects. Notably, KG NP retained a relatively high TPC and antioxidant activity compared to other NP formulations, indicating possible differences in formulation efficiency or improved stability of active compounds.

##### Collagenase Inhibitory Activity

Collagenase, a zinc-dependent multidomain enzyme, plays a key role in collagen degradation, contributing to skin wrinkling and the aging process. Therefore, inhibiting collagenase activity represents a strategic approach to delaying collagen breakdown and maintaining skin integrity [19]. This study evaluated the collagenase inhibitory activity of kratom leaf extracts and their corresponding NP formulations, using EGCG as a positive control and mitragynine as a bioactive marker for comparison.

**Figure 7:**
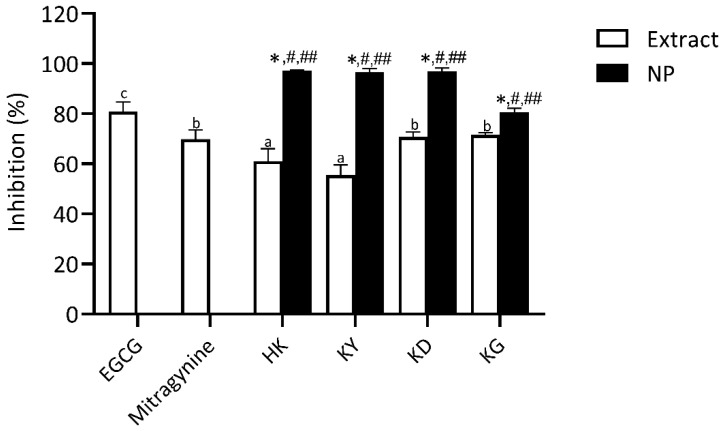
Collagenase inhibitory activity of kratom leaf extracts and their NP formulations compared to epigallocatechin gallate (EGCG) and mitragynine. All samples were tested at a concentration of 1 mg/mL. Bars represent mean ± SD (*n* = 3). Different letters indicate significant differences among the extracts (*p* < 0.05, one-way ANOVA with Tukey’s test). Symbols from pairwise *t*-tests (*p* < 0.05): * vs. corresponding extract; *#* vs. mitragynine; *##* vs. EGCG.

As shown in Figure 7, all tested samples demonstrated varying degrees of collagenase inhibition. EGCG demonstrated the highest inhibitory activity (80.8 ± 3.9%), while mitragynine showed a moderate inhibitory activity (69.9 ± 3.7%), which was significantly lower than that of EGCG but notably higher than the HK and KY extracts. Interestingly, KD and KG showed the most pronounced collagenase inhibition (70.7 ± 1.0 and 71.6 ± 0.8%, respectively) among the crude extracts, with values statistically comparable to mitragynine. These findings are particularly promising for anti-aging applications, as collagenase activity directly contributes to collagen degradation and the loss of skin structural integrity associated with visible signs of skin aging [19]. Previous studies have reported that the collagenase inhibitory effect of EGCG is primarily due to its polyphenolic structure. The hydroxyl groups of polyphenols play a crucial role by forming hydrogen bonds with the backbone amide and functional groups of the collagenase enzyme [20]. Additionally, hydrophobic interactions between the benzene rings of polyphenols and the enzyme may induce conformational changes, resulting in enzyme inactivation [21].

However, the collagenase inhibition observed with kratom leaf extracts may involve mechanisms distinct from the direct enzyme binding typically associated with polyphenols. The indole alkaloids from *M. speciosa*, particularly mitragynine, are known to exert their effects primarily through anti-inflammatory and antioxidant mechanisms [8,22]. Mitragynine has been shown to suppress the cyclooxygenase–prostaglandin E2 (COX-PGE2) signaling pathway, resulting in the down regulation of matrix metalloproteinase-1 (MMP-1), a collagenase responsible for collagen degradation [23,24]. This indirect mechanism suggests that mitragynine and kratom extracts can offer protection against collagen degradation and support anti-aging benefits, even in the absence of direct enzymatic inhibition. Moreover, kratom leaf extracts contain a diverse array of bioactive compounds, including flavonoids, polyphenols, and terpenoids, all known to contribute to various pharmacological activities [25]. The antioxidant properties demonstrated in this study suggest that these compounds may act synergistically, enhancing the overall inhibitory effect on collagenase activity. Such synergism may further support the potential utility of kratom leaf extracts in anti-aging skincare applications.

In the NP formulations, collagenase inhibitory activity significantly improved across all kratom leaf extracts. Notably, HK NP had the highest activity (97.2 ± 0.3%), which was not only significantly higher than its crude extract counterpart but also comparable to the inhibitory activity of EGCG and mitragynine. This suggests that the NP formulation of the HK extract achieves inhibition levels similar to those of well-established anti-aging agents. KY NP, KD NP, and KG NP also showed a strong inhibition in the range of 80.56 ± 1.60–97.23 ± 0.29%, all significantly higher than the activities of their respective crude extracts, EGCG, and mitragynine. The substantial enhancement in collagenase inhibition observed in the NP formulations may result from the improved dispersibility of particular active components upon encapsulation. These findings highlight the potential of kratom-derived nanoparticles—particularly HK NP—as promising candidates for anti-aging skincare applications targeting collagen breakdown and skin matrix preservation.

##### Elastase Inhibitory Activity

**Figure 8:**
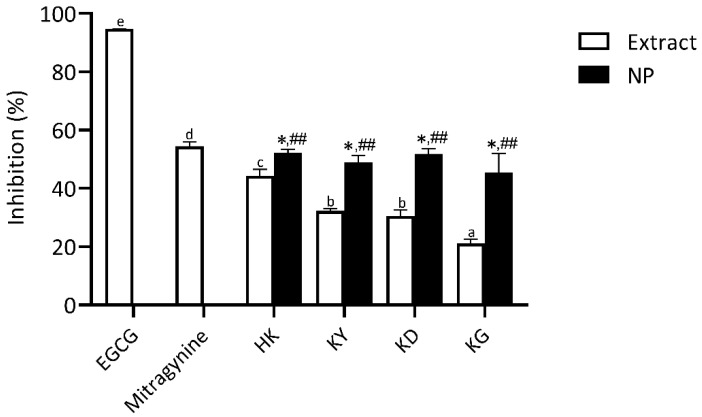
Elastase inhibitory activity of kratom leaf extracts and their NP formulations compared to epigallocatechin gallate (EGCG) and mitragynine. All samples were tested at a concentration of 1 mg/mL. Bars represent mean ± SD (*n* = 3). Different letters indicate statistically significant differences among extracts (*p* < 0.05, one-way ANOVA with Tukey’s test). Symbols from pairwise *t*-tests (*p* < 0.05): * vs. corresponding extract; *##* vs. EGCG.

Elastin, a key component of the extracellular matrix (ECM), is essential for maintaining skin elasticity and structural integrity. Elastase, a serine protease, hydrolyzes elastin and other ECM proteins, and its dysregulation is closely associated with skin aging processes [26]. In this study, the elastase inhibitory activity of kratom leaf extracts and their NP formulations was evaluated and compared to EGCG and mitragynine. As shown in Figure 8, all tested samples exhibited varying degrees of elastase inhibition. EGCG demonstrated the highest elastase inhibitory activity (94.7 ± 0.1%), significantly greater than all other tested samples. Mitragynine exhibited moderate inhibition (54.5 ± 1.5%). Among the crude extracts, HK showed the highest elastase inhibition (44.3 ± 2.3%), followed by KY, KD, and KG, which demonstrated a progressively lower inhibition.

Since elastase is a serine protease responsible for elastin degradation, its inhibition is critical for preserving skin firmness and elasticity [27]. Natural elastase inhibitors such as catechin and EGCG are well-documented, with polyphenols playing a significant role in this activity [28,29]. This study found that mitragynine, an indole alkaloid, and kratom leaf extracts exhibited a moderate but significant elastase inhibition; however, their efficacy was considerably lower than that of EGCG. The observed elastase inhibitory activity of kratom extracts may be influenced by their distinct composition of bioactive compounds, including indole alkaloids and polyphenols. While polyphenols are well known for their direct enzyme inhibition through specific molecular interactions, indole alkaloids may contribute to elastase regulation through alternative mechanisms, such as the suppression of inflammatory pathways and oxidative stress [30,31,32]. These complementary effects highlight the potential of kratom extracts in anti-aging formulations, particularly when combined with other compounds that provide direct enzyme-inhibitory activity.

All NP formulations exhibited significantly higher elastase inhibitory activity than their respective crude extracts, with inhibition levels ranging from approximately 45.46 ± 6.53% to 52.19 ± 1.20%. However, no significant differences were observed among the NP formulations, indicating that the improvement primarily arises from nanoencapsulation itself, which enhances the delivery and effectiveness of active compounds. Although the inhibition levels of the NP formulations remained lower than EGCG, their enhanced activity compared to crude extracts highlights the potential of nanotechnology in improving the efficacy of kratom extracts for dermal anti-aging applications targeting elastase inhibition.

##### Hyaluronidase Inhibitory Activity

**Figure 9:**
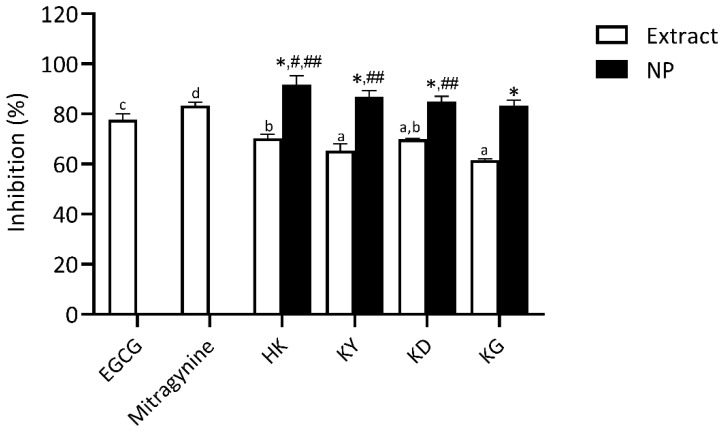
Hyaluronidase inhibitory activity of kratom leaf extracts and their NP formulations compared to epigallocatechin gallate (EGCG) and mitragynine. All samples were tested at a concentration of 1 mg/mL. Bars represent mean ± SD (*n* = 3). Different letters indicate significant differences among extracts (*p* < 0.05, one-way ANOVA with Tukey’s test). Symbols from pairwise *t*-tests (*p* < 0.05): * vs. corresponding extract; *#* vs. mitragynine; *##* vs. EGCG.

Hyaluronic acid (HA) is a vital component of the extracellular matrix (ECM), playing a key role in maintaining skin hydration and moisture balance. Enhancing HA levels primarily involves inhibiting hyaluronidase activity, as this enzyme is responsible for HA degradation [4]. Therefore, the use of effective hyaluronidase inhibitors represents a targeted strategy to preserve HA content and support skin hydration. The hyaluronidase inhibitory activity of kratom leaf extracts and their NP formulations was evaluated and compared to EGCG, a well-established positive control, and mitragynine, the principal bioactive marker compound. As shown in Figure 9, all samples exhibited varying degrees of hyaluronidase inhibition. Mitragynine (83.33 ± 1.35%) and EGCG (77.75 ± 2.42%) exhibited high levels of inhibitory effects, both significantly greater than those of the kratom leaf extracts. The strong activity of mitragynine suggests a possible interaction with the enzyme, potentially preventing the degradation of hyaluronic acid, a crucial component for maintaining skin hydration and firmness The crude kratom extracts, including HK, KY, KD, and KG, demonstrated moderate hyaluronidase inhibition (61.56 ± 0.51–70.31 ± 1.66%), with no statistically significant difference among several samples, indicating a relatively similar bioactive compound profile. The notable hyaluronidase inhibitory effect of mitragynine observed in this study may be mechanistically associated with its well-documented anti-inflammatory properties, which may also contribute indirectly to the regulation of hyaluronidase activity.

Following NP formulations, all kratom leaf extracts showed significantly enhanced hyaluronidase inhibitory activity. HK NP demonstrated the highest inhibitory activity (91.67 ± 3.56%), exceeding that of its corresponding extract and statistically comparable to mitragynine. Other NP samples—KY NP (86.83 ± 2.48%), KD NP (84.98 ± 2.06%), and KG NP (83.23 ± 2.34%) also showed marked improvements over their respective extracts. Other NP samples—KY NP (86.3 ± 2.5%), KD NP (84.9 ± 2.1%), and KG NP (83.2 ± 2.3%) also showed marked improvements over their respective extracts. These results suggest that nanoencapsulation effectively preserves or enhances the hyaluronidase-inhibiting activity of the bioactive constituents, highlighting their potential for skincare applications aimed at retaining skin moisture by protecting hyaluronic acid from enzymatic degradation.

The significant inhibition of hyaluronidase by mitragynine may also be attributed to its anti-inflammatory properties. Hyaluronidase not only degrades hyaluronic acid but also promotes inflammation by generating low-molecular-weight hyaluronic acid fragments that act as damage-associated molecular patterns (DAMPs). These fragments can trigger inflammation and tissue degradation [33]. Mitragynine, the main indole alkaloid in *Mitragyna speciosa*, has been shown to inhibit pro-inflammatory mediators such as TNF-α, IL-1β, and COX-2, while suppressing NF-κB signaling, all of which are closely involved in inflammation and aging processes [34,35]. Similarly, ginkgoside B dimethyl ester, an indole alkaloid N-glycoside derived from *Ginkgo biloba*, has been reported to protect dermal fibroblasts by reducing MMP-1 expression, restoring collagen levels, and inhibiting MAPK and Akt signaling pathways [36]. These findings suggest that mitragynine may exert protective effects on the skin through similar anti-inflammatory and anti-degradative mechanisms.

The anti-aging related enzyme activities indicate that while mitragynine contributes to enzyme inhibition, especially collagenase and hyaluronidase, its activity alone does not fully explain the observed effects. EGCG, used as a positive control, exhibited superior elastase and collagenase inhibition, reinforcing its role as a potent anti-aging standard. Therefore, the anti-enzyme effects in kratom extracts and NPs are likely involving not only mitragynine but also polyphenolic compounds.

##### Cytotoxicity Evaluation on HaCaT Cells

**Figure 10:**
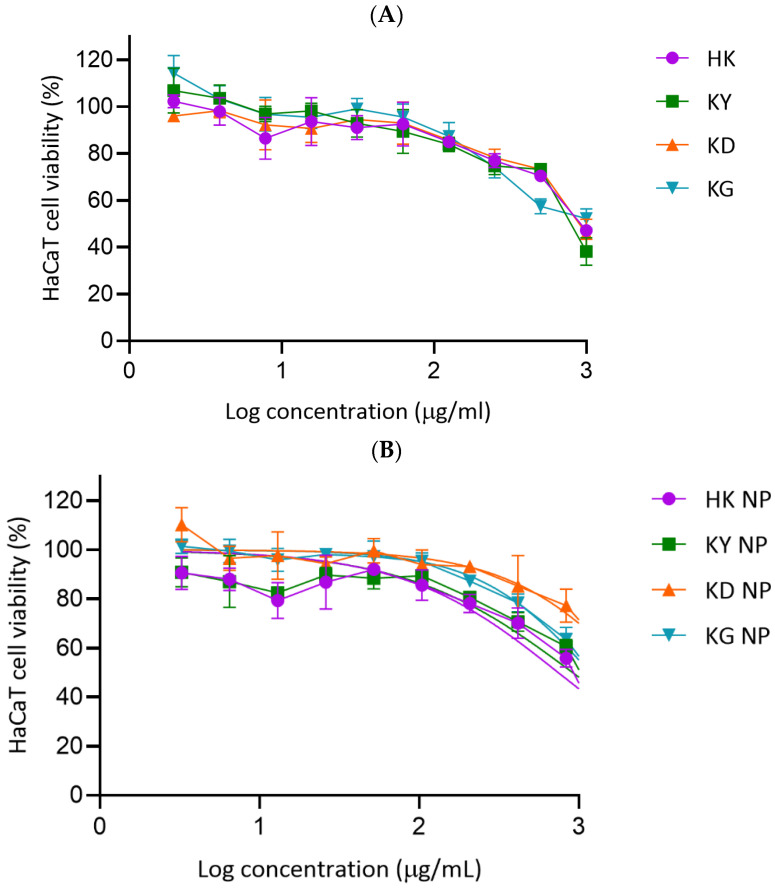
Cytotoxicity of kratom extracts and their NPs formulations in HaCaT cells. (**A**) Cell viability following exposure to increasing concentrations of crude extracts (HK, KY, KD, and KG). (**B**) Cell viability following exposure to nanoparticle formulations (HK NP, KY NP, KD NP, and KG NP). Cell viability was measured using the MTT assay and expressed as a percentage relative to untreated control cells. Data represent mean ± SD (*n* = 3).

The cytotoxicity of kratom leaf extracts and their NP formulations was evaluated using the MTT assay in HaCaT human keratinocyte cells across a range of log concentrations. As shown in Figure 10A,B, both crude extracts and NP formulations demonstrated a dose-dependent reduction in cell viability, with varying IC_50_ values. Among the crude extracts, HK and KD exhibited the highest IC_50_ values (1265.20 ± 325.44 and 1252.57 ± 430.55 µg/mL, respectively), indicating a lower cytotoxicity. KG and KY extracts showed moderately lower IC_50_ values (896.70 ± 37.65 and 762.55 ± 15.34 µg/mL, respectively), suggesting a comparatively higher cytotoxicity. For the NP formulations, KD NP exhibited the highest IC_50_ value (1232.00 ± 140.50 µg/mL), followed by KG NP (718.33 ± 118.47 µg/mL), indicating an improved cytocompatibility relative to their respective crude extracts. In contrast, HK NP showed a reduced IC_50_ (447.67 ± 77.88 µg/mL), suggesting a slightly higher cytotoxicity than its crude extract. KY NP had an intermediate IC_50_ value of 550.87 ± 33.80 µg/mL. Overall, most extracts and NP formulations maintained over 80% cell viability at concentrations below 100 µg/mL. The findings suggest that kratom samples, particularly in nanoparticle form, are generally well tolerated by HaCaT cells at moderate concentrations. The improved IC_50_ values observed for KD NP and KG NP support their potential for safer application in skin-related formulations.

The IC_50_ values derived from the cytotoxicity assay against HaCaT keratinocyte cells were statistically analyzed using Tukey’s multiple comparisons test. Comparisons among the crude extracts, HK, KD, KY, and KG, revealed no statistically significant differences (*p* > 0.05 for all pairwise comparisons), indicating comparable cytotoxicity profiles among these extracts. When comparing the nanoparticle formulations, significant differences were observed between KD NP and both KY NP and HK NP, suggesting that KY NP and HK NP exerted stronger cytotoxic effects than KD NP. However, no significant differences were found among the other nanoparticle groups, indicating a relatively similar cytotoxicity among KY NP, HK NP, and KG NP. When comparing each extract with its corresponding nanoparticle formulation, only HK NP demonstrated a significantly lower IC_50_ value compared to the HK extract (*p* = 0.0031), suggesting an enhanced cytotoxicity through nanoparticle delivery. In contrast, the differences between extract and nanoparticle forms for KD, KY, and KG were not statistically significant (*p* > 0.05). These findings suggest that nanoparticle encapsulation selectively enhanced the cytotoxic potential of the HK extract, while other formulations showed comparable activity to their crude counterparts.

##### Preliminary Physical and Sensory Evaluation of Kratom Nanoparticle-Loaded Gels

**Figure 11:**
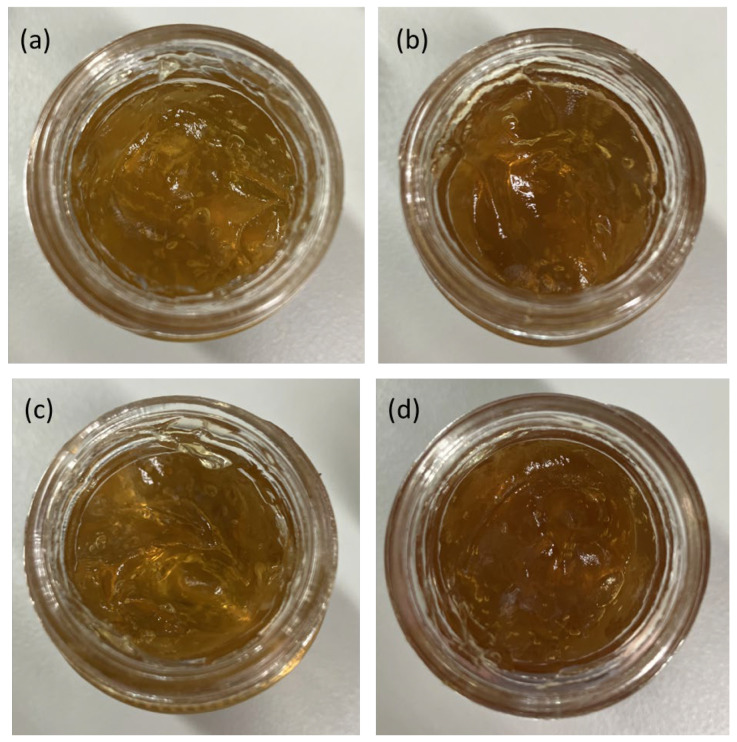
Macroscopic appearance of kratom NP-loaded gel formulations. Photographs of gels containing (**a**) HK NPs, (**b**) KY NPs, (**c**) KD NPs, and (**d**) KG NPs. All formulations exhibited a clear to translucent amber color with uniform consistency, indicating successful incorporation of kratom nanoparticles into the gel matrix. No phase separation, precipitation, or visual signs of instability were observed.

All four kratom NP-based gel formulations containing HK NP, KY NP, KD NP, and KG NP exhibited a clear to translucent, brownish appearance, indicating good clarity and the uniform dispersion of the NPs within the gel matrix (Figure 11). The assessment of physical and sensory attributes such as texture, spreadability, and tackiness was performed subjectively by the researchers through visual inspection and manual handling. While this preliminary evaluation suggested acceptable characteristics for topical application, no formal sensory panel or instrumental texture analysis was conducted, and no human volunteer testing was performed.

##### Characterization of Kratom Nanoparticle-Loaded Gels and Their Stability

Physical characteristics

After six heating/cooling cycles in the stability assay, the physical characteristics of all four kratom NP-loaded gel formulations had favorable physical properties. Each gel appeared clear and brownish, with a uniform texture and no visible phase separation, indicating good homogeneity and proper dispersion of active ingredients.

pH Measurement

All freshly prepared kratom nanoparticle-loaded gel formulations demonstrated near-neutral to slightly alkaline pH values. KD NP and KY NP gels both exhibited the initial highest pH, averaging 7.83 ± 0.05 and 7.83 ± 0.03, respectively, followed by KG NP gel at 7.56 ± 0.04 and HK NP gel at 7.27 ± 0.06. After undergoing six cycles of heating and cooling in a stability assay, a slight increase in pH was observed in most formulations. KD NP and KY NP gels again recorded the highest pH values at 7.88, while HK NP gel increased marginally to 7.33 ± 0.18. KG NP gel maintained a stable pH of 7.56 ± 0.09. These findings suggest overall stability in pH across all formulations, with only minimal changes following thermal stress.

The pH values of the formulations ranged from 7.27 to 7.83, which are near-neutral and within the acceptable range for topical application (pH 5.3–7.6). These values suggest a low likelihood of skin irritation and support the potential for safe dermal use. Moreover, the low standard deviation among repeated measurements indicates good formulation consistency and batch reproducibility.

Rheological Evaluation

Upon fresh preparation, the KG NP-loaded gel displayed the highest viscosity (12.602 ± 0.294 Pa·s), followed closely by the KY NP-loaded gel (12.291 ± 0.038 Pa·s) and HK NP (11.898 ± 0.139 Pa·s). The KD NP-loaded gel had the lowest initial viscosity at 11.518 ± 0.072 Pa·s. After six cycles of heating and cooling in the stability assay, a slight reduction in viscosity was noted across all samples, though the relative order remained unchanged. The KG NP gel still showed the highest viscosity (11.674 ± 0.097 Pa·s), followed by HK NP (11.197 ± 0.163 Pa·s), KY NP (11.073 ± 0.233 Pa·s), and KD NP (10.719 ± 0.261 Pa·s). The blank gel formulation exhibited intermediate viscosity values at both time points (11.885 ± 0.225 Pa·s initially and 11.577 ± 0.071 Pa·s after thermal cycling), indicating that the presence of the kratom extract influenced the rheological behavior.

Collectively, these findings indicate that the kratom NP gel formulations maintained desirable physical characteristics under the studied conditions. The combination of stable appearance, acceptable pH, and consistent viscosity suggests that these formulations are suitable candidates for topical delivery. Nevertheless, accelerated and long-term stability testing under standard recommended storage conditions would be essential to confirm their robustness for commercial application.

## 3. Conclusions

Among the four *Mitragyna speciosa* extracts, KG exhibited the highest TPC and TFC. Antioxidant activities varied depending on the assay, with the KY extract showing the strongest DPPH and FRAP activities, while KG led in ABTS scavenging. KG showed the highest collagenase inhibition, whereas the HK extract was most effective against elastase and hyaluronidase. Upon nanoparticle formulation, all activities were enhanced. HK NP demonstrated the strongest DPPH and ABTS scavenging capacities as well as the greatest inhibitory effects against collagenase, elastase, and hyaluronidase. The kratom NP-loaded gels also exhibited desirable physicochemical properties, including suitable pH, consistent viscosity, and good visual stability, supporting their suitability for topical formulations.

## 4. Materials and Methods

### 4.1. Materials

Gallic acid, Griess reagent, DPPH (2,2-diphenyl-1-picrylhydrazyl), TPTZ (2,4,6-Tris(2-pyridyl)-s-triazine), and ABTS (2,2′-azino-bis(3-ethylbenzothiazoline-6-sulfonic acid)) were obtained from Sigma-Aldrich (St. Louis, MO, USA). Chemicals, including absolute ethanol, dimethyl sulfoxide (DMSO), sodium bicarbonate, sodium nitrate, acetic acid, and sodium hydroxide, were supplied by RCI Labscan (Bangkok, Thailand). Iron(III) chloride hexahydrate and 37% hydrochloric acid were purchased from Qrec Chemical Co., Ltd. (Auckland, New Zealand). The Folin–Ciocalteu phenol reagent, aluminum chloride, sodium acetate trihydrate, ferrous sulfate heptahydrate (purity ≥ 99%), and potassium persulfate were sourced from Loba Chemie (Mumbai, India). Ascorbic acid and poloxamer 407 were acquired from Chanjao Longevity Co., Ltd. (Bangkok, Thailand). Dulbecco’s Modified Eagle’s Medium (DMEM, high glucose), fetal bovine serum (FBS), penicillin-streptomycin, and trypsin-EDTA were provided by Gibco (Waltham, MA, USA). Clostridium histolyticum collagenase (EC.3.4.23.3), porcine pancreatic elastase (PE–E.C.3.4.21.36), bovine testis hyaluronidase (E.C.3.2.1.3.5), a synthetic peptide of N-[3-(2-furyl) acryloyl]-Leu-Gly-Pro–Ala (FALGPA), N-succinyl-Ala–Ala–Ala–p-nitroanilide (AAAPVN), epigallocatechin gallate (EGCG, 98% purity), and hyaluronic acid were purchased from Sigma-Aldrich (St. Louis, MO, USA).

### 4.2. Methods

#### 4.2.1. Extraction of Kratom (*Mitragyna speciosa* (Korth.) Havil.)

**Figure 12:**
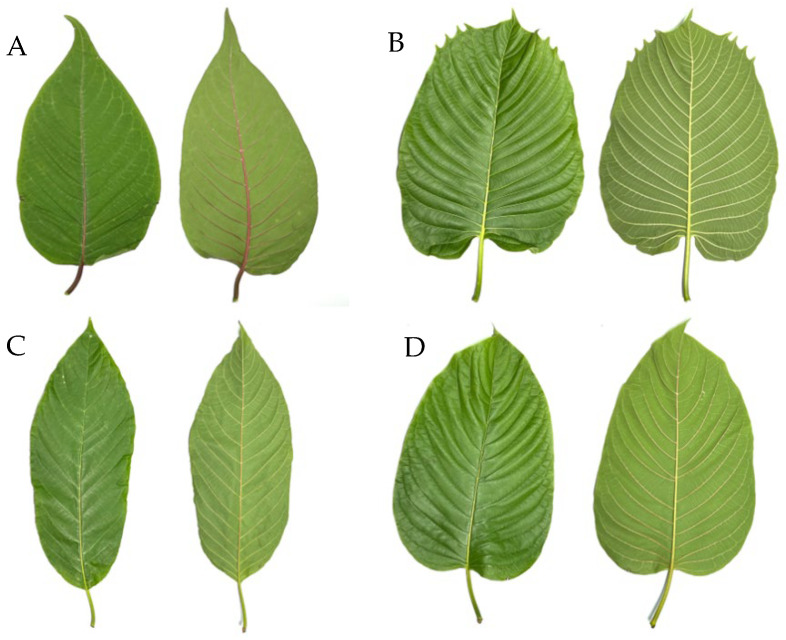
The genetic variation of *Mitragyna speciosa* (kratom) analyzed in this study included the following accessions: (**A**) Kan Daeng (KD), (**B**) Hang Kang (HK), (**C**) Tai Bai-yao (KY), and (**D**) Kan Keaw (KG).

Various kratom leaf samples were collected for this study, including Kan Daeng (KD), Yak Yai or Hang Kang (HK), Tai Bai-yao (KY), and Tang Kua or Kan Keaw (KG) (Figure 12). The kratom samples were collected from reputable plantations in Thailand, specifically from Surat Thani Province (9.00546° N, 98.94927° E for KD and 9.03296° N, 98.96132° E for KG) and Pathum Thani Province (14.10881° N, 100.42706° E for KY and HK). The plant materials were identified based on morphological characteristics, namely, leaf, flower, and fruit structures, by comparison with the taxonomic descriptions reported by Ngernsaengsaruay et al. [37]. To preserve thermolabile phytochemicals in kratom leaves, drying was conducted at 50 °C, a temperature shown to be effective in preventing the degradation of key alkaloids such as mitragynine. This is supported by Orio et al., who reported that extraction at 50 °C did not significantly compromise the alkaloid content in *Mitragyna speciosa* leaf extracts [38]. Dohárszky et al. demonstrated that ultrasound-assisted extraction at 50 °C yielded a high mitragynine content, comparable or superior to lower temperatures and substantially better than higher-temperature Soxhlet extraction, which caused alkaloid degradation [39]. Once dried, the leaves were ground into fine powder using an electric grinder. A total of 400 g of the powdered sample was subjected to extraction with 4000 mL of ethanol (10:1 solvent-to-sample ratio) using a Soxhlet apparatus (Haberg, Germany) for 20 h. The solvent was subsequently removed under reduced pressure using a rotary evaporator. The obtained crude extracts were stored in amber bottles and kept refrigerated for further experimental use.

#### 4.2.2. Formulation and Characterization of Kratom Nanoparticles

Kratom nanoparticles were prepared using the solvent replacement method. Briefly, kratom leaf extracts from KD, HK, KY, and KG (800 µL of 0.125 g/2 mL in 95% ethanol), centrifuged at 13,000 rpm for 3 min to precipitate the undissolved extract residue, were introduced dropwise into a mixture of 13.5 mL of 0.1% *w*/*v* poloxamer 407 and 1.5 mL of polyethylene glycol 400 (PEG 400) [12,16]. PEG 400 was used to enhance the solubilization of hydrophobic phytochemicals and improve nanoparticle stabilization. The addition was performed at a constant rate of 1 mL/h under continuous magnetic stirring at 800 rpm, and the system was maintained at room temperature (30 °C) to facilitate nanoparticle formation via rapid solvent diffusion and precipitation of the bioactive constituents. The resulting formulations were labeled as KD NP, HK NP, KY NP, and KG NP. To produce blank NPs, the same method was followed, omitting the kratom leaf extract. Kratom NPs were analyzed for particle size, polydispersity index (PDI), and zeta potential using a Zetasizer (Malvern Instruments, Worcestershire, UK). The dynamic light scattering (DLS) measurements were conducted using water as the dispersant, with a refractive index of 1.330 and a viscosity of 0.8872 cP at 25 °C. The material refractive index was set to 1.590 with an absorption value of 0.010, as selected from the instrument database. Measurements were performed at a backscatter angle of 173° using a DTS0012 cell. The samples were allowed to equilibrate for 120 s prior to measurement. To ensure an accurate particle size analysis and avoid multiple scattering, the formulations were ten-fold diluted with deionized water prior to measurement.

#### 4.2.3. Total Phenolic Content Determination

The total phenolic content in kratom extracts (122.07–62,500 µg/mL) and kratom NPs (6.45–3300 µg/mL) was determined using a microplate-based Folin–Ciocalteu assay. Briefly, samples (50 µL) were added to 96-well plates, followed by 100 µL of 10% Folin–Ciocalteu reagent [40]. After 4 min incubation, 50 µL of 10% sodium carbonate was added, and the reaction was left in the dark at room temperature for 60 min. Absorbance was measured at 765 nm. Gallic acid (1.95–1000 µg/mL) served as the standard, and results were expressed as gallic acid equivalent (GAE).

#### 4.2.4. Total Flavonoid Content Determination

Kratom extracts (122.07–62,500 µg/mL), kratom NPs (6.45–3300 µg/mL), and standards (quercetin: 1.95–1000 µg/mL; EGCG: 1.95–1000 µg/mL) were dispensed (50 µL/well) into 96-well plates. The concentration ranges for the extracts and nanoparticles differed due to differences in formulation constraints. For the nanoparticle formulations, lower concentrations were used because of the limited drug loading efficiency, which necessitated controlling the concentration to maintain an optimal particle size, polydispersity index, and physical stability. In contrast, higher concentrations of crude extracts were required to achieve measurable activity levels in the assay. To each well, 30 µL of 5% sodium nitrate was added, incubated for 5 min, followed by 50 µL of 2% aluminum chloride [40]. After a 6 min reaction time, 50 µL of 1 N sodium hydroxide was added, and the plate was incubated for 10 min. The absorbance was recorded at 510 nm. The flavonoid content was quantified using standard calibration curves and expressed as the quercetin equivalent (QE) for kratom extracts and EGCG equivalent (EGCGE) for kratom nanoparticle formulations.

#### 4.2.5. HPLC Quantification of Bioactive Markers

The HPLC method for mitragynine analysis was adapted from a previously published report [41]. The HPLC analysis of the bioactive marker mitragynine was performed using a Waters Alliance e2695 system (Milford, MA, USA) equipped with a quaternary pump, autosampler, column thermostat, and photodiode array (PDA) detector. Data processing was conducted using the Empower 3 software (Waters, Milford, MA, USA). Chromatographic separation was achieved on a BDS Hypersil C18 analytical column (4.6 × 250 mm, 5 µm; Thermo Scientific, Waltham, MA, USA). The mobile phase consisted of an isocratic mixture of acetonitrile and 10 mM ammonium formate (65:35, *v*/*v*), delivered at a flow rate of 1.0 mL/min. The column was maintained at ambient temperature. Detection was carried out at 225 nm, which is the optimal wavelength for mitragynine. The injection volume for all standard and sample solutions was 5 µL per run. The mitragynine standard was used for calibration and quantification. Retention time and peak area were compared against the standard curve generated from known concentrations of mitragynine to determine its content in the samples.

#### 4.2.6. DPPH Radical Scavenging Assay

The antioxidant capacity of kratom extracts, kratom NPs, mytragynine (1.95–1000 µg/mL), and the antioxidant standard (ascorbic acid: 1.95–1000 µg/mL) was assessed using the DPPH radical scavenging assay. Samples (100 µL) were pipetted into 96-well microplates, followed by an equal volume (100 µL) of 0.1 mM DPPH solution. After a 30 min incubation in the dark at room temperature, absorbance at 517 nm was recorded [42]. Radical scavenging activity was calculated using the following equation:

$$\mathrm{DPPH}\;\mathrm{Scavenging}\;(\%)=[1-\left(\frac{{Abs}_{sample}}{{Abs}_{control}}\right)]\times 100$$

where ${Abs}_{control}$ represents the absorbance of the control reaction (reagent with DI water), and ${Abs}_{sample}$ is the absorbance of the test sample (extracts or nanoparticles with reagent).

#### 4.2.7. ABTS Radical Scavenging Assay

Briefly, 20 µL of each test sample (kratom extracts, kratom NPs, mytragynine, and ascorbic acid at 1.95–1000 µg/mL) were combined with 180 µL of 0.1 mM ABTS•+ solution in 96-well microplates. After 15 min incubation in the dark at room temperature, absorbance was measured at 734 nm [42]. Antioxidant activity was calculated as follows:

$$\mathrm{ABTS}\;\mathrm{Scavenging}\;(\%)=[1-\left(\frac{{Abs}_{sample}}{{Abs}_{control}}\right)]\times 100$$

where ${Abs}_{control}$ represents the absorbance of the control reaction (reagent with DI water), and ${Abs}_{sample}$ is the absorbance of the test sample (extracts or nanoparticles with the reagent).

#### 4.2.8. Ferric Reducing Antioxidant Power Assay

The FRAP assay was used to assess the reducing power of kratom extracts, kratom NPs, mytragynine, and ascorbic acid (1.95–1000 µg/mL). A 20 aliquot of each sample was mixed with 180 µL of freshly prepared FRAP reagent (acetate buffer, 300 mM, pH 3.6; ferric chloride, 20 mM; and TPTZ, 10 mM in a 10:1:1 ratio). After 30 min of incubation at 37 °C, absorbance at 595 nm was measured [42]. A standard curve was generated using ferrous sulfate (9.8–5000 µM) for calculating FRAP values.

#### 4.2.9. Collagenase Inhibitory Assay

The collagenase inhibitory activity of kratom extracts, kratom NPs, mytragynine, and EGCG was determined following the method described by Preedalikit et al. [29]. A collagenase enzyme solution was prepared using a 50 mM tricine buffer (pH 7.5) supplemented with 400 mM sodium chloride and 10 mM calcium chloride. The substrate used was the synthetic peptide FALGPA, which mimics the structural features of collagen. To prepare the substrate solution, FALGPA was dissolved in the same tricine buffer to achieve a final concentration of 2 mM. Test samples (10 µL at 1 mg/mL of kratom extracts, kratom NPs, mytragynine, and EGCG) were mixed with 40 µL of a collagenase enzyme solution and allowed to incubate at room temperature for 15 min. The enzymatic reaction was initiated by adding 50 µL of the substrate solution. The absorbance was then monitored immediately at 340 nm in kinetic mode using a microplate reader (Synergy H1, Agilent Technologies, Santa Clara, CA, USA). The percentage of collagenase inhibition was calculated using the following formula:

$$\mathrm{Collagenase}\;\mathrm{inhibition}\;(\%)=\frac{{Abs}_{control}-{Abs}_{sample}}{A{bs}_{control}}\times 100$$

where ${Abs}_{control}$ represents the absorbance of the control reaction (containing DI water, enzyme, and substrate), and ${Abs}_{sample}$ corresponds to the absorbance of the test sample (containing extract or nanoparticles, enzyme, and substrate).

#### 4.2.10. Elastase Inhibitory Assay

The elastase inhibitory activity was evaluated based on the method by Preedalikit et al. [29], using N-succinyl-Ala-Ala-Ala-p-nitroanilide (AAAPVN) as a specific substrate. Briefly, 50 µL of the kratom extracts, kratom NPs, mytragynine, or EGCG at a concentration of 1 mg/mL were pre-incubated with 25 µL of elastase enzyme solution (2 mg/mL, prepared in 100 mM Tris-HCl buffer, pH 8.0) at room temperature for 20 min. Following this, 25 µL of the AAAPVN substrate (4.4 mM in Tris-HCl buffer) was added to initiate the enzymatic reaction. The change in absorbance at 410 nm was monitored immediately in kinetic mode using a microplate reader (Synergy H1, Agilent Technologies, Santa Clara, CA, USA). The percentage inhibition of elastase activity was calculated using the following equation:

$$\mathrm{Elastase}\;\mathrm{inhibition}\;(\%)=\frac{{Abs}_{control}-{Abs}_{sample}}{A{bs}_{control}}\times 100$$

where ${Abs}_{control}\;$ is the absorbance of the control reaction (comprising DI water, elastase enzyme, and substrate), and ${Abs}_{sample}\;$ is the absorbance of the test reaction containing the extract or nanoparticles, enzyme, and substrate.

#### 4.2.11. Hyaluronidase Inhibitory Assay

The inhibitory effect of the samples on hyaluronidase activity was assessed using a turbidimetric method based on the procedure by Preedalikit et al. [29]. This assay evaluates the extent of hyaluronic acid degradation catalyzed by hyaluronidase enzyme. Residual, undegraded hyaluronic acid forms a turbid precipitate, and thus, the degree of turbidity correlates inversely with enzyme activity. Kratom extracts, kratom NPs, mitragynine, and EGCG (50 µL each) were pre-incubated with 100 µL of hyaluronidase solution (2 mg/mL) at 37 ± 5 °C for 10 min. After pre-incubation, 100 µL of 0.03% (*w*/*v*) hyaluronic acid in 300 mM phosphate buffer (pH 5.35) was added, and the mixture was incubated again at 37 ± 5 °C for 45 min. To precipitate the remaining hyaluronic acid, 1 mL of acetic albumin solution (prepared from sodium acetate, acetic acid, and bovine serum albumin, pH 3.75) was added. The mixture was left to stand at room temperature for 10 min to allow turbidity to develop. Absorbance was then measured at 600 nm using a microplate reader (Synergy H1, Agilent Technologies, Santa Clara, CA, USA). The percentage of hyaluronidase inhibition was calculated using the following equation:

$$\mathrm{Hyaluronidase}\;\mathrm{inhibition}\;(\%)=\left(\frac{{Abs}_{sample}}{{Abs}_{control}}\right)\times 100$$

where ${Abs}_{control\;}$ refers to the absorbance of the control reaction containing DI water, hyaluronic acid, and acetic albumin solution (without enzyme), and ${Abs}_{sample\;}$ represents the absorbance of the reaction containing the extract or nanoparticles, enzyme, hyaluronic acid, and acetic albumin solution.

#### 4.2.12. Cell Culture and Cytotoxicity Assay

The HaCaT cell line was cultured in high-glucose DMEM supplemented with 10% FBS and 1% penicillin-streptomycin. Cultures were maintained at 37 °C in a humidified 5% CO_2_ incubator and subcultured every three days using 0.25% trypsin-EDTA. For the cytotoxicity assay, HaCaT keratinocytes were seeded in 96-well plates at a density of 2 × 10^3^ cells per well and incubated for 24 h to allow cell attachment. Cells were then treated with kratom extracts and NPs for 24 h. After treatment, MTT solution (0.5 mg/mL, 100 µL) was added to each well and incubated at 37 °C for 2 h. Formazan crystals formed by viable cells were dissolved in 100 µL of dimethylsulfoxide (DMSO), and absorbance was measured at 550 nm using a microplate reader. The IC_50_ values were calculated using GraphPad Prism v8.0 (La Jolla, CA, USA), and cell viability was determined using the following formula:

$$\mathrm{Cell}\;\mathrm{viability}\;(\%)=\left(\frac{{Abs}_{sample}}{{Abs}_{control}}\right)\times 100$$

where ${Abs}_{control\;}$ refers to the absorbance of the untreated cells and ${Abs}_{sample\;}$ represents the absorbance of the treated cells.

#### 4.2.13. Preparation of the Kratom Nanoparticle-Loaded Gel

A 100 g batch of the nanoparticle-loaded gel was prepared using carbomer-based formulation. Purified water (83 g) was transferred into a beaker and stirred at 1200 rpm using a magnetic stirrer. Carbomer 980 (1.5 g) was gradually sprinkled into the water with continuous gentle stirring with a glass rod until a homogeneous dispersion was achieved. Glycerin (5 g) was then added and mixed thoroughly. Triethanolamine was added dropwise to adjust the pH until a clear and viscous gel base was formed. Subsequently, the previously prepared kratom NP suspension (10 g) was incorporated and mixed until uniformly dispersed. Finally, phenoxyethanol (0.5 g) was added as a preservative and the mixture was stirred until a homogeneous gel was obtained. The finished gel was filled into suitable containers for storage.

#### 4.2.14. Characterization of Kratom Nanoparticle-Loaded Gel

##### pH Measurement

The pH of the kratom nanoparticle-loaded gel was measured in triplicate at room temperature using a calibrated pH meter (Thermo Fisher Scientific, Waltham, MA, USA). To evaluate the formulation’s stability, pH measurements were also conducted after subjecting the samples to a heating/cooling stability test consisting of six complete cycles. Each cycle involved storing the gel for 24 h at 45 °C followed by 24 h at 4 °C.

##### Rheological Evaluation

The rheological properties of the kratom nanoparticle-loaded gel were assessed using a rotational rheometer (Anton Paar MCR 102e Rheometer, Graz, Austria) equipped with a plate–plate configuration. Measurements were performed at a constant shear rate of 100 s^−1^ while gradually increasing the temperature from 25 °C to 35 °C at a rate of 1 °C/min. Rheograms were generated using Rheocompass software version 1.33. To investigate thermal stability and viscoelastic consistency under stress conditions, the rheological behavior was also evaluated before and after the six-cycle heating/cooling stability test (as described above).

#### 4.2.15. Statistical Analysis

All experimental data are expressed as the mean ± standard deviation (SD) from three independent experiments (*n* = 3). A statistical analysis was performed using a one-way analysis of variance (ANOVA), followed by Tukey’s multiple comparisons post hoc test to determine significant differences between groups. A *p*-value of less than 0.05 was considered statistically significant. All analyses were conducted using GraphPad Prism version 8.02 (GraphPad Software, La Jolla, CA, USA).

The English language of this manuscript was reviewed and refined using AI-based language tools (ChatGPT (GPT-4), OpenAI, San Francisco, CA, USA) to improve clarity, grammar, and readability prior to submission.

## Figures and Tables

**Table 1:** Comparative Pearson correlation analysis between phytochemical content and the IC_50_ values of kratom extracts and nanoparticle formulations.

Comparison	Kratom Extract		Kratom NPs	
	Pearson r	*p*-Value	Pearson r	*p*-Value
TPC vs. DPPH	0.5802	0.4198	−0.7984	0.2016
TPC vs. ABTS	−0.9755	0.0245	−0.9308	0.0692
TPC vs. FRAP	0.8492	0.1508	0.8566	0.1434
TFC (EGCG) vs. DPPH	0.6272	0.3728	−0.7891	0.2109
TFC (EGCG) vs. ABTS	−0.9856	0.0144	−0.8581	0.1419
TFC (EGCG) vs. FRAP	0.8713	0.1287	0.9106	0.0894
TFC (quercetin) vs. DPPH	0.6274	0.3726	−0.7889	0.2111
TFC (quercetin) vs. ABTS	−0.9856	0.0144	−0.8577	0.1423
TFC (quercetin) vs. FRAP	0.8712	0.1288	0.9108	0.0892

## Data Availability

The original contributions presented in this study are included in the article. Further inquiries can be directed to the corresponding author.
